# Identification of metal sensitization in sarcoid-like metal-exposed patients by the MELISA® lymphocyte proliferation test — a pilot study

**DOI:** 10.1186/s12995-016-0101-1

**Published:** 2016-04-12

**Authors:** Elizabeth Fireman, Amir Bar Shai, Yifat Alcalay, Noa Ophir, Shmuel Kivity, Vera Stejskal

**Affiliations:** Laboratory for Pulmonary and Allergic Diseases, Tel Aviv Medical Center, 6 Weizman Street, Tel Aviv, 6423906 Israel; Environmental and Occupational Medicine, Sackler School of Medicine, Tel Aviv University, Tel Aviv, Israel; Wenner Gren Center, University of Stockholm, Stockholm, Sweden; Research Centre for Toxic Compounds in the Environment, Masaryk University, Brno, CzechRepublic

**Keywords:** Granuloma, Inorganic exposure, Occupational diseases

## Abstract

**Background:**

Pulmonary function is often affected by the inhalation of metal particles. The resulting pathology might trigger various lung diseases, e.g., parenchymal lung fibrosis and granulomatous lung disorders. We previously demonstrated that 6 % of tissue-proven sarcoid patients had a positive beryllium lymphocyte proliferation test (BeLPT), thus correcting the diagnosis to chronic beryllium disease. The aim of this study was to examine if MEmory Lymphocyte Immnuno Stimulation Assay (MELISA®), currently used for non-pulmonary diseases, can identify metals other than beryllium that can also trigger sensitization and induce granulomatous disease.

**Methods:**

This pilot study included 13 sarcoid-like patients who underwent MELISA®. Eleven patients also underwent BeLPT. Biopsy samples were tested for metal content by scanning electron microscope. Eleven study patients had been exposed to metals at the workplace and 2 had silicone implants.

**Results:**

Two patients who had undergone BeLPT were positive for beryllium. MELISA® detected 9 patients (9/13, 69 %) who were positive for at least one of the tested metals: 4 reacted positively to nickel, 4 to titanium, 2 to chromium, 2 to beryllium, 2 to silica, and one each to palladium, mercury and lead.

**Conclusion:**

It is proposed that MELISA® can be exploited to also identify specific sensitization in individuals exposed to inhaled particles from a variety of metals.

## Background

Metals are known to cause a number of different pathological conditions, including pulmonary disease [1, 2]. The inhalation of metal dust can cause a variety of lung diseases, such as parenchymal lung fibrosis and granulomatous lung disorders [3, 4]. Granulomatous inflammation and hypersensitivity pneumonitis are associated with the inhalation of metal dust and fumes as well as with mycobacterial or fungal infections [4].

Increased industrial uses of beryllium and consequent occupational beryllium exposure resulted in an epidemic of chronic beryllium disease (CBD) in the United States and other countries [5]. The delayed type hypersensitivity induced by beryllium serves as a model for immunologically driven granulomatous lung disease [6, 7]. We had previously demonstrated that 6 % of tissue-proven sarcoid patients who had a positive beryllium lymphocyte proliferation test (BeLT) had been erroneously diagnosed as having sarcoidosis and not CBD [8]. The rate of granulomatous lung disease in workers exposed to the metals other than beryllium is unknown. Because of the relative paucity of case reports on other metals, the incidence might either be low or underreported.

It was recently shown that lymphocyte proliferation tests can be useful in assessing occupational sensitization [9]. Several case reports demonstrated increased lymphocyte proliferation to titanium [10], aluminum [11, 12] chromium and nickel [13], the latter as a side effect following hip arthroplasty. MEmory Lymphocyte Immnuno Stimulation Assay (MELISA®) is a lymphocyte proliferation test currently used in the setting of non-pulmonary diseases. We conducted a pilot study to determine whether MELISA® is also effective in identifying sensitization to a number of selected metals in a cohort of exposed sarcoid patients with lung granulomatous diseases who had been exposed to various substances at the workplace and in the environment.

## Methods

The study cohort was comprised of 13 sarcoid patients who were referred to our Institute of Pulmonary and Allergic Diseases to undergo occupational exposure assessment. The characteristics of the patients are listed in Table 1. Eleven patients had been exposed to toxic airborne substances at the workplace and two patients developed granulomatous diseases after ruptured silicone implants (Table 1). Each participant filled in a questionnaire on demographic and occupational parameters and a general health profile. They all underwent a lung biopsy which confirmed the diagnosis of noncaseating granulomas. Sarcoidosis was diagnosed by a clinical and roentgenological evaluation or a positive transbronchial biopsy. All subjects gave written informed consent to be enrolled in this investigation, and the study was approved by the Tel-Aviv Medical Center Institutional Ethics Committee.

**Table 1:** Patient characteristics

Number	Gender	Age (yrs)	Exposure route
1	Male	70	Military industry
2	Male	61	Metal industry
3	Female	63	Jewelry industry
4	Male	58	Aviation industry
5	Male	70	Welding
6	Male	63	Welding
7	Male	63	Military industry
8	Male	45	Dental technician
9	Male	50	Dental technician
10	Female	40	Dental technician
11	Female	45	Teaching
12	Female	35	Silicone implants
13	Female	40	Silicone implants

MELISA® is an optimized lymphocyte transformation test that was described in depth elsewhere [14]. For the current work, blood samples were drawn into tubes containing citrate in the Tel Aviv lab and sent for analysis to the MGD Laboratory in Geneva, Switzerland. The blood was delivered by courier after overnight transport not exceeding 24 h and at ambient temperature. The choice of metals for testing was based on information derived from the questionnaire, which was delivered to the laboratory together with the blood sample.

BeLPT was performed within 24 h of venipuncture according the method of Mroz et al. [15]. Results were expressed as a stimulation index (SI), which is the ratio of the counts per minute of radioactivity in cells stimulated with beryllium salts divided by the counts per minute for un-stimulated cells. The normal ranges for the SI are laboratory-dependent and based on the mean peak SI plus 3 standard deviations for unexposed subjects. SIs higher than those values were considered as a positive response to beryllium. To fulfill the criteria for a positive beryllium-specific response, at least two or more elevated SIs occurring at any of the BeSO4 concentrations tested must have been present. An SI >2.5 was considered elevated.

**Fig. 1:**
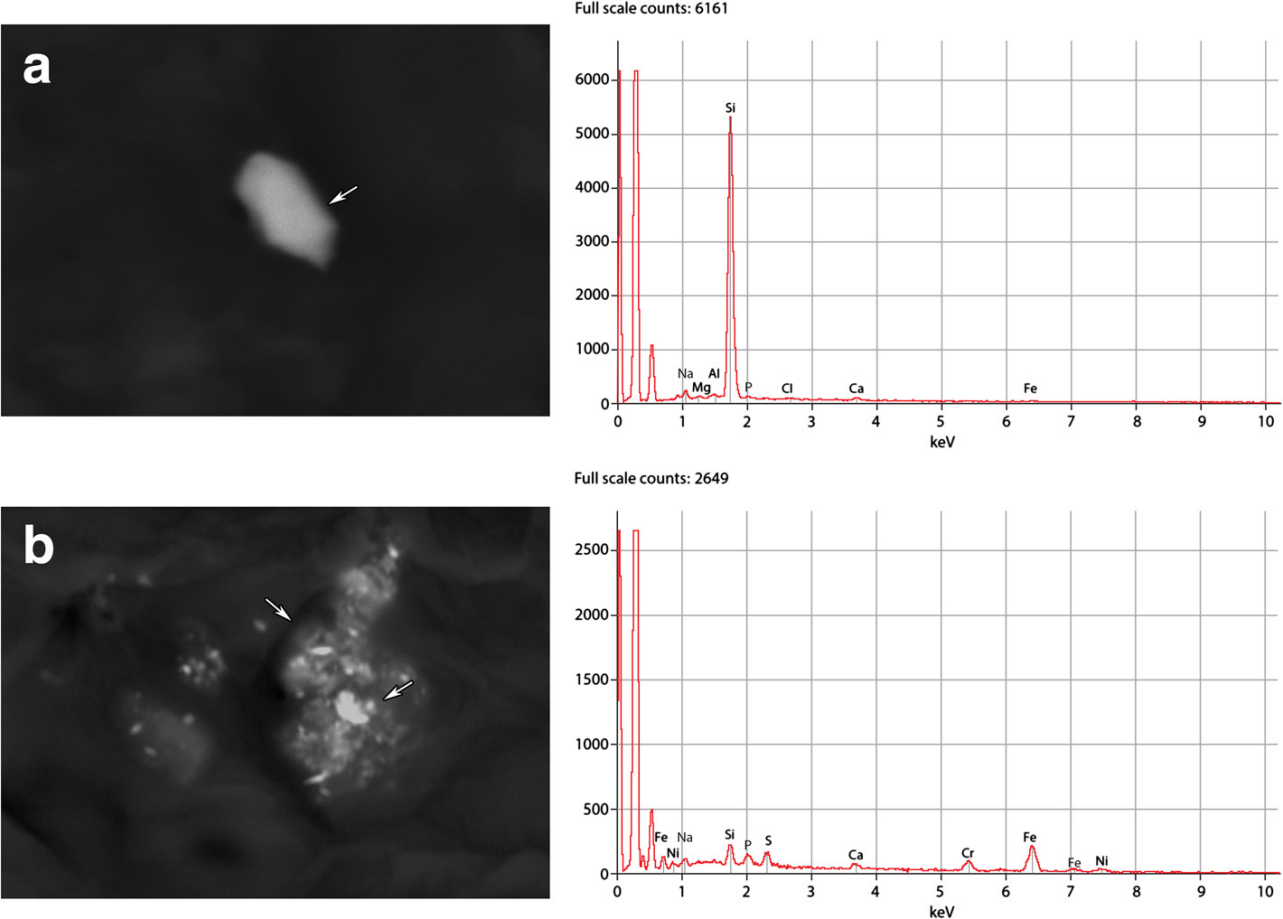
A representative analysis of lung tissue biopsy by SEM. **a** Particles containing Al, Si, and Fe. **b** Particles containing Ni, Si, Fe, and Cr

Mineralogical studies were performed on the paraffin block of the lung biopsy. A sample from a paraffin block was processed for electron microscopic studies by embedding it in Epon. Thin sections were cut and lightly contrasted by uranyl acetate The chemical composition of selected specimens was investigated by counting 500 particles (>0.8 μm in diameter) by X-ray analysis using a JEOL 840 scanning electron microscope (SEM) equipped with a Link 10,000 energy-dispersive system (EDS). The spectrometer of the EDS system separates the elements according to energy rather than wavelength. A petrographic microscope was also used to identify the minerals. A representative SEM analysis of the lung tissue biopsy of patient #2 (Fig. 1).

## Results

A total of 13 sarcoid patients were enrolled in this pilot study. MELISA® was targeted to identify specific metals according to their occupational questionnaire and type of exposure. SEM was performed in lung biopsies and showed noncaseating granulomas in 12/13 patients. All the exposed workers but not the silicone implanted women also underwent BeLPT. The tested metals and the test results are shown in Table 2.

**Table 2:** Results of scanning electron microscopy (SEM), the beryllium lymphocyte proliferation test BeLPT and MELISA®

Pt.	Diagnosis	Metals tested by SEM	Metals tested by MELISA®	MeLPT	BeLPT	Exposure route
1	SA; NCG (+)	Fe, Ti, Al, Si	Al, Si, Ti	(-)	Be(-)	Military industry
2	SA; NCG (+)	Si, Cr, Ni, Fe	Cr, Fe, Ni, Si, Ti, Be	Ti(+)	Be(-)	Metalworks
3	SA; NCG (+)	Si, Cr, Ni, Fe, Mo	Cr, Au, Hg, Pa, Ti, Ni, Mo	Ti(+) Pa(+)	Be(-)	Jewelry industry
4	SA; NCG (+)	Au, Zn, Si, Al,	Al, Au, Hg, Pa, Si, Ti, Zn, Be	Hg(+)	Be(-)	Aviation industry
5	SA; NCG (−)	Al, Ag, Cu	Ag, Cu, Al	(-)	Be(-)	Welding
6	SA; NCG (+)	Al, Si, Fe, Zn	Hg, Ni, Si, Ti, Zn, Pb, Ti	Ti(+) Pb(+)	Be(-)	Welding
7	SA; NCG (+)	Al, Si Fe	Al, Au, Fe, Si, Ti, Be	Ti(+) Be(+)	Be(-)	Military industry
8	CBD NCG (+)	(−)	Fe, Ni, Ti, Be	(-)	Be(+)	Dental technician
9	CBD NCG (+)	Al, Si Cr, Fe, Ni	Al, Cr, Fe, Ni, Si, Be	Si(+)Fe (+)Cr(+) Ni(+) Be(+)	Be(+)	Dental technician
10	SA; NCG (+)	Si, Cr, Co, Fe, Ti, Ni, W, Mn	Cr, Co, Fe, Mn, Ni, Si, Ti,	(-)	Be(-)	Dental technician
11	SA; NCG (+)	Fe, Cr, Ni, Si	Cr, Hg, Fe, Ni, Si, Ag, Sn	Si(+)Cr(+) Ni(+)	Be(-)	Teacher
12	SA; NCG (+)	Si, Au, Cr, Fe	Au, Hg, Ni, Pa, Pl, Si	Ni(+)	ND	Silicone implants
13	SA; NCG (+)	Si	Au, Ni, Pa, Pl, Si	Ni(+)	ND	Silicone implants

*Pt* patient #, *SA* sarcoidosis, *NCG* non-caseating granulomas, *ND* not done, *Al* aluminum, *Cu* copper, *Si* silica, *Fe* iron, *Ti* titanium, *Cr* chrome, *Ni* nickel, *Au* gold, *Mo* molybdenum, *Zn* zinc, *Hg* mercury, *Mn* manganese, *Co* cobalt, *Pb* lead, *W* tungsten, *Pa* palladium

**Fig. 2:**
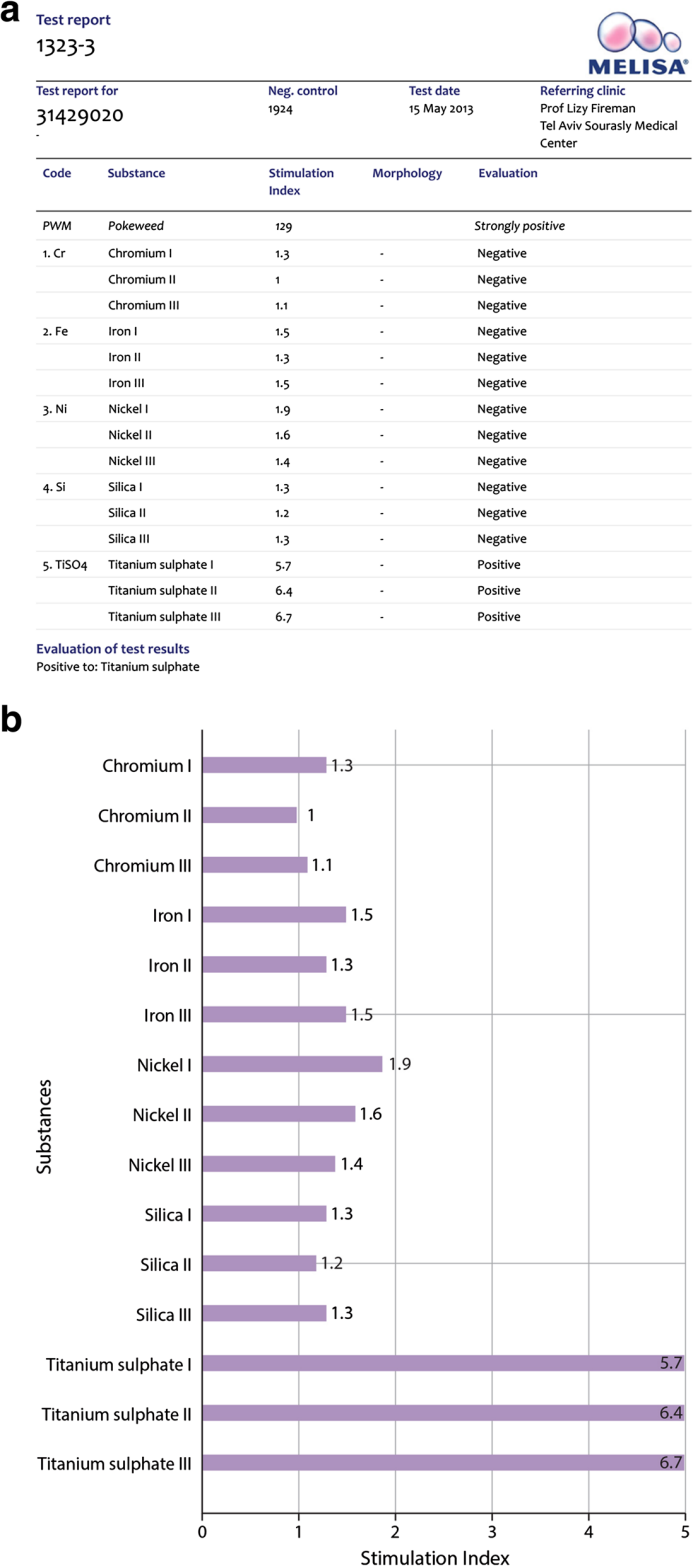
A representative report of MELISA®. **a** Positive control (PWM, popweed mitogen) and negative results for Cr, Fe, Ni and Si but positive for Ti. **b** Negative results for Cr, Fe, Ni and Si but positive for Ti

MELISA® detected 9 patients (9/13, 69 %) who were positive for at least one of the tested metals: 4 reacted positively to nickel, 4 to titanium, 2 to chromium, 2 to beryllium, 2 to silica, and one each to palladium, mercury and lead. Both patients who developed lung granulomatous disease following the rupture of silicone implants had a positive MELISA® result to nickel (Table 2). A representative report for a MELISA® evaluation is shown in Fig. 2.

BeLPT detected two of the 13 patients as being positive to beryllium and 11 as being negative. Seven of the 11 BeLPT-negative patients (63.6 %) were positive for at least one of the tested metals. Three of the 11 workplace-exposed patients were dental technicians and they were exposed to many metals used in dentistry, but only one of them responded positively to chrome, nickel and beryllium. One of the remaining two was negative to all tested metals and the other responded positively to beryllium only on the Be-LPT evaluation.

## Discussion

Accurate diagnosis of sarcoidosis requires a careful inquiry about any potential exposure to both organic and inorganic allergens. Several epidemiologic investigations have suggested that it is an occupational or infectious (e.g., viruses, *Borrelia burdorfori*, and *Propionibacterium acnes*) [16] clustering disease. The fact that sarcoidosis generally develops in early adulthood suggests that both infectious and noninfectious causative exposures occur in working-age individuals as they enter the workforce [17]. Other studies have postulated that exposure to granuloma-inducing allergens at the workplace may cause sarcoidosis. For example, a clustering of cases was detected in communities with lumbering or wood milling as the principal local industry [18]. Others have found clustering of cases among mechanics, postal workers [18], firefighters [19] and healthcare workers [20].

We were the first to document a case of CBD in Israel in a dental technician who was first diagnosed as having sarcoidosis [21]. The comprehensive environmental anamnesis was instrumental in correcting the diagnosis to CBD. Motivated by this case, we performed a prospective study and recalled patients from our outpatient clinic who had been diagnosed as having sarcoidosis. We reevaluated their occupational exposure history and found that 3/47 patients (6 % of all patients diagnosed with sarcoidosis) with a positive occupational exposure to beryllium and a positive BeLPT had CBD and had been misdiagnosed as having sarcoidosis.8 These results emphasize the vital importance of taking an occupational history together with administering the BeLPT, the cornerstone of both medical surveillance and diagnosis of beryllium sensitization and disease. In the same context there is a need to take occupational history to other type of exposures and to include other lymphocyte proliferation tests to other metals other than beryllium. Sensitizers and other case reports had been published showed increased lymphocyte proliferation in response to titanium [10], aluminum [11, 12], chrome and nickel [13].

To the best of our knowledge, this is the first report on patients with biopsy-proven granulomatous disease in whom deposition of metals in the lungs was demonstrated by evidence of multiple metal sensitization on MELISA® simultaneously with the SEM analysis. The metals were chosen for testing according to occupational history, the responses to an overall metal exposure questionnaire, and the metals identified in the lung tissue biopsy.

We did not find a good correlation between SEM metal analysis and the results of lymphocyte proliferation testing may be due to the fact that the analysis is done randomly on 3–4 places on the biopsy surface [22], and either does not represent the total content of the metals in the tissue or there is an insufficient concentration of metal for detection with SEM [23]. Another explanation is that may be that like for beryllium CD4 T cell responding in the blood is determined by the relative proportion of memory T cell subsets, which is influenced by the degree of target organ inflammation [24]. Moreover, it was recently shown that anti-HLA-DP and anti-LFA-1 antibodies are present in individuals with sensitization to beryllium and that they may eliminate proliferation ability and cytokine production [25].

On the other hand, we had demonstrated a very strong response to beryllium in the BeLPT test of a patient with post-traumatic shrapnel deposited in the chest wall. The concentration of beryllium in the shrapnel was very low: it was undetectable by SEM and only detected by ion mass spectrometry [26]. Similarly, Stejskal recently reported that a very low concentration of beryllium in the gold crown of a tooth of a fibromyalgia patient induced a strong MELISA®-positive reaction to beryllium [27].

It was concluded that the most frequent metals that cause granulomatous disease, such as aluminum, barium, cobalt, copper, gold, nickel, titanium, and zirconium, should be tested by a widely applicable and sensitive lymphocyte proliferation test, such as MELISA®, in sarcoid patients who are exposed to metals at the workplace. Other metals for evaluation should be selected from the individual’s occupational and environmental exposure history. Some metals, such as aluminium, silica and mercury, might play a dual role in inflammatory reactions: in addition to their function as antigens, they also have immunomodulatory properties and act as adjuvants in increasing the immune response to others inhaled metals.

## Conclusions

In conclusion, results of this pilot study serve to emphasize the vital importance of taking a comprehensive occupational history in the clinical evaluation of patients suspected of having sarcoidosis.

The rate of granulomatous lung disease in workers exposed to the metals other than beryllium is unknown. Because of the relative paucity of case reports on metals other than beryllium, the incidence might either be underreported. Identification of metal sensitization in sarcoid-like metal-exposed patients by the MELISA® lymphocyte proliferation test may have a crucial role to avoid misdiagnosis in this population.
